# FOXO1-regulated lncRNA LINC01197 inhibits pancreatic adenocarcinoma cell proliferation by restraining Wnt/β-catenin signaling

**DOI:** 10.1186/s13046-019-1174-3

**Published:** 2019-04-26

**Authors:** Jing Ling, Fan Wang, Chuan Liu, Xiao Dong, Ying Xue, Xuebing Jia, Weifeng Song, Qi Li

**Affiliations:** 1 Department of Oncology, Shanghai General Hospital, Shanghai Jiao Tong University School of Medicine, Shanghai, 200080 China; 2 Shanghai Key Laboratory of Pancreatic Diseases, Shanghai, 200080 China

**Keywords:** LINC01197, FOXO1, Pancreatic ductal adenocarcinoma, Proliferation, Wnt/β-catenin signaling

## Abstract

**Background:**

Recent studies have revealed that numerous oncogenic long non-coding RNAs (lncRNAs) play pivotal roles in pancreatic ductal adenocarcinoma (PDAC) progression, but little is known about tumor-suppressive lncRNAs in PDAC. This study was conducted to evaluate the function of tumor-suppressive LINC01197 in PDAC progression and investigate the detailed mechanisms.

**Methods:**

LncRNA microarray was used to identify differentially expressed lncRNAs in FOXO1-overexpressing PANC1 cells. LINC01197 expression was evaluated by quantitative PCR, Northern blotting, and fluorescence in situ hybridization. The Cancer Genome Atlas database was used to analyze the prognostic role of LNC01197 in PDAC. A luciferase reporter assay was performed to confirm the interaction between LNC01197 and FOXO1. The biological function of LINC01197 was evaluated by colony formation assay in vitro and in an animal subcutaneous tumorigenesis experiment and Ki67 staining in vivo. RNA-pulldown, western blotting, RNA immunoprecipitation assay, and co-immunoprecipitation were further performed to determine the molecular mechanism of LNC01197 and β-catenin in the Wnt pathway.

**Results:**

We found that a FOXO1-related lncRNA, LINC01197, was significantly decreased in PDAC malignant tissues and that its low expression predicted poor prognosis. Moreover, LINC01197 was mainly localized in the nucleus and inhibited PDAC cell proliferation both in vitro and in vivo. Mechanistically, LINC01197 was found to bind to β-catenin and inhibit Wnt/β-catenin signaling activity by disrupting β-catenin binding to TCF4 in PDAC cells.

**Conclusions:**

The novel FOXO1/LINC01197/β-catenin axis was dysregulated during PDAC progression. Our study provides insight into the mechanisms of LINC01197 in PDAC and reveal a potential target for PDAC clinical therapy and prognostic prediction.

## Background

Pancreatic ductal adenocarcinoma (PDAC), commonly referred to as pancreatic cancer, is a highly aggressive malignant tumor and is one of the leading causes of cancer-associated mortality worldwide [1, 2]. Patients with PDAC typically show insidious early symptoms and the pancreas is anatomically covered by gastrointestinal and other organs, making it difficult to detect early lesions by conventional imaging techniques. When patients are diagnosed with pancreatic cancer, 25% of cases are locally advanced and 60% are unresectable or metastatic, with most patients experiencing significant pain [3]. Although numerous studies have evaluated the detection and management of PDAC, the five-year relative survival rate remains at only 5%. Therefore, further investigation of the molecular basis of PDAC pathogenesis is urgently required.

The Forkhead transcription factor family member FOXO1 is a multifunctional transcription factor [4] that regulates the transcription of downstream target genes by post-translational modification, participating in reactive oxygen species and DNA repair, apoptosis, cell cycle regulation, and other processes in various tumors, particularly in digestive malignancies [5–7]. Activation of FOXO1 results in upregulation of the cyclin-dependent kinase inhibitor p27KIP1 and down-regulation of D-type cyclins, thereby arresting cells at G1 [8]. Activated FOXO1 also triggers apoptosis in many cancer cell lines by regulating various proapoptotic proteins, including Fas ligand, TRAIL, and Bim [9]. Our previous studies demonstrated that FOXO1 is significantly down-regulated in PDAC tissues, which is correlated with cancer cell stemness in PDAC [10, 11]. However, no long non-coding (lncRNA) directly regulated by FOXO1 in PDAC has been reported.

LncRNAs are a class of pervasive RNAs involved in various biological functions and play emerging roles in cancer development including transcriptional regulation, RNA processing, translational control, epigenetic modification, and posttranslational modification [12]. LncRNAs are related to nearly all aspects of gene regulation and protein function. A previous study revealed the common archetypes of molecular functions executed by lncRNAs as signals, decoys, guides, and scaffolds [13]. Recent studies reported that lncRNAs such as AFAP1-AS1, Linc00675, MALAT-1, lncRNA PVT1, ENST00000480739, and HOTTIP may play multiple roles, such as oncogenic or tumor-suppressive roles, in PDAC cells [14–19]. LINC01197, also referred to NONHSAT050194.2 in the NONCODE database, is a newly identified non-coding RNA which has not been investigated previously in cancer.

Abnormal activation of Wnt/β-catenin signaling has been observed in numerous solid tumors, including PDAC [20, 21]. Wnt/β-catenin signaling plays a pivotal role in the physiological processes of cells and can target numerous oncogenes such as MYC, CCND1, CD44, and MMP26, all of which contribute to tumor progression [22–24]. Many factors have been identified to interact with the β-catenin-TCF4/LEF-1 complex, which then recruits the transcription factors Brg1 and CREB-binding protein to initiate Wnt-targeted gene expression, such as lncRNA CRNDE, Nemo-like kinase, and APC [25–27]. In this study, we found that LINC01197 binds to β-catenin and inhibits the activity of Wnt/β-catenin signaling by disrupting β-catenin binding to TCF4.

## Methods

### Patient tissues and ethics statement

Eighteen fresh tumor tissues and matched adjacent tissues were collected from patients with pathologically and clinically confirmed PDAC from Shanghai General Hospital. All human tumor tissues were collected after obtaining written informed consent from the patients. The Institutional Review Board of Shanghai General Hospital approved the use of the tumor samples and animals in this study.

### Cell culture

HPNE, AsPC1, BxPC3, and PANC1 were purchased from American Type Culture Collection (Manassas, VA, USA). All cells were maintained under standard culture conditions (37 °C, 5% CO_2_) in culture medium recommended by American Type Culture Collection. All cells were characterized/authenticated by DNA typing at the Shanghai Jiao Tong University Analysis Core.

### RNA isolation and quantitative real-time PCR

Total RNA was purified from PDAC and adjacent tissues or cells using TRIzol (Invitrogen, Carlsbad, CA, USA) according to the manufacturer’s protocol. RNA (1 μg) was reverse-transcribed using SuperScript Reverse Transcriptase III (Invitrogen). Quantitative real-time PCR was performed using SYBR green Supermix (Applied Biosystems, Foster City, CA, USA) in an ABI 7300 PCR system. GAPDH was used as a reference gene. Primers used in this study are shown in Table 1.

**Table 1:** sequence of primers

Name	Sequence (5′-3′)
Linc01197 primer	F: CCAAATCCTCGGTGCTGTGA
	R: TGCCTCTGTACGCAGATTCC
GAPDH primer	F:AGCCTCAAGATCATCAGCAATGCC
	R: TGTGGTCATGAGTCCTTCCACGAT
CCND1 primer	F: TCCTCTCCAAAATGCCAGAG
	R: GGCGGATTGGAAATGAACTT
CD44 primer	F:CTGCCGCTTTGCAGGTGTA
	R: CATTGTGGGCAAGGTGCTATT
MYC	F: CGTCCTCGGATTCTCTGCTC
	R: CTTCGCTTACCAGAGTCGCT
MMP26	F:TCGGAATGGGACAGACCTACT
	R:TCAAAGGGGTCACATTGCTCC

### Western blotting

Tissues and cells were lysed in WB/IP lysis buffer (P0013, Beyotime, Shanghai, China) and nuclear proteins were extracted using lysis buffer (P0028, Beyotime); all procedures were conducted according to the manufacturer’s protocol. Subsequently, the cell lysates were boiled in 5X SDS-PAGE loading buffer for 10 min and then resolved by 8% SDS-PAGE and transferred to a nitrocellulose membrane. The following antibodies were used in this study: FOXO1 (Cell Signaling Technology, Danvers, MA, USA), GAPDH (Proteintech, Rocky Hill, NJ, USA), TCF4 (Cell Signaling Technology), β-catenin (Cell Signaling Technology), and Lamin A/B (Cell Signaling Technology). Bound antibodies were visualized with an ECL kit (Thermo Fisher Scientific, Waltham, MA, USA).

### Northern blotting

A total of 20 μg of total RNA was electrophoresed on a 10% NovexTM TBE-Urea Gel (Invitrogen) and transferred to a Hybond-N+ membrane using a semi-dry electroblotter at 400 mA for 30 min, followed by UV crosslinking. Oligo DNA probes (10 ng each) were labeled with gamma [32P]-ATP using a MEGALABEL Kit (Invitrogen). Hybridization was performed in hybridization buffer (10% SDS, 10% dextran sulfate, 1 M NaCl, 0.5 mg/mL sonicated salmon sperm DNA) at 65 °C overnight. The membranes were washed twice in 2x SSC and 0.5% SDS at room temperature for 30 min and in 0.2x SSC and 0.5% SDS at 65 °C for 30 min. Next, the signals were visualized with a BAS-3000 image-analyzer (GE Healthcare, Little Chalfont, UK).

### Subcellular fractionation

Cytoplasmic and nuclear fractions were isolated by NE-PER™ Nuclear and Cytoplasmic Extraction Reagents (Thermo Fisher Scientific) and collected using an RNeasy Midi Kit (Qiagen, Hilden, Germany) to determine the cellular localization of LINC01197. All procedures were conducted according to the manufacturer’s protocol. RNAs were extracted from each fraction and subjected to quantitative real-time polymerase chain reaction (qRT-PCR) analysis to determine the levels of LINC01197, GAPDH, and U1.

### Stable cell line generation

To generate cell lines with stable ectopic expression of FOXO1 and LINC01197, vectors containing full-length FOXO1 and LINC01197 were purchased from GeneCopoeia (Rockville, MD, USA). Cells were transfected using lipofectamine 2000 (Invitrogen) with the vectors following the manufacturer’s instructions. The supernatant medium containing the virus was collected by centrifugation to remove cellular contaminants. The resulting viruses were used to infect the indicated cells, and transfected cells were then selected using 2 μg/mL puromycin for 2 weeks. Alterations in FOXO1 and LINC01197 in these cells were confirmed by western blotting and qPCR before further analysis.

### siRNA treatment

Short interfering RNAs (siRNAs; Sigma, St. Louis, MO, USA) were used to knock down FOXO1 and LINC01197 and their sequences are shown in Table 2. Cells were transfected with 100 nM siRNAs or with 100 nM RNAi negative control using Lipofectamine 2000 (Invitrogen). Alterations in LINC01197 in these cells were confirmed by western blotting and qPCR before further analysis.

**Table 2:** The target sequences of FOXO1 and Linc01197 sequence of primers

si97#1	ATGTTCTGAGACCAGTTTAAA
si97#2	GACCTAAAGCTAGTCAAATAT
siFOXO1#1	CATGGACAACAACAGTAAATT
siFOXO1#2	TTTGATAACTGGAGTACATTT

### Animal experiment

The indicated stable cell lines (1 × 10^6^) were subcutaneously injected into the right flank of 5 BALB/c (nu/nu) mice in each group. Tumor size was measured once per week and mice were sacrificed to analyze the tumor burden after 4 weeks and the tumor volume was calculated with the following formula: V = (length × width^2^)/2″. All procedures of animal experiments were performed in accordance with The Animal Care and Use Committee of Shanghai Jiao Tong University School of Medicine.

### Immunohistochemical staining

Tissue samples were stained to identify and measure Ki67 levels. The tumors were detected with primary monoclonal probes for Ki67 overnight at 4 °C. After incubation with a suitable second antibody, the tissue microarrays were treated with diaminobenzidine and counterstained with hematoxylin. All tissues were observed and photographed (10X) with a microscope (Carl Zeiss, Oberkochen, Germany).

### Fluorescence in situ hybridization analysis

HPNE cells were used for RNA fluorescence in situ hybridization (FISH) analysis. Nuclear and cytosolic fraction separation was performed using a PARIS kit (Life Technologies, Carlsbad, CA, USA), and RNA FISH probes were designed and synthesized by Bogu according to the manufacturer’s instructions. Briefly, the cells were fixed in 4% formaldehyde for 15 min and then washed with phosphate-buffered saline (PBS). The fixed cells were treated with pepsin and dehydrated with ethanol. The air-dried cells were further incubated with 40 nM of the FISH probe in hybridization buffer. After hybridization, the slide was washed, dehydrated, and mounted with Prolong Gold Antifade Reagent with DAPI for detection. The slides were visualized for immunofluorescence with an Olympus microscope (Tokyo, Japan).

### Colony formation assays

Cells were trypsinized and resuspended in 1.5 mL of 0.35% agarose and poured onto 1.5 mL of a 0.7% agarose bed in 6-well plates. The cells (1000/well) were incubated for nearly 3 weeks at 37 °C and 5% CO_2_. Colonies were fixed and stained with 0.5% crystal violet and the number of colonies was counted.

### Luciferase reporter assays

For promoter identification, the luciferase reporter PGL3-3XDBE plasmid was purchased from Promega (Madison, WI, USA), and the promoter of LINC01197 was inserted into the PGL3-Basic plasmid between the BamH1 and EcoR1 sites. For Wnt canonical signaling luciferase assays, the TCF/LEF (Top flash) and mutated TCF/LEF (Fop flash) reporter plasmids were purchased from Promega. Indicated cells were seeded into 96-well plates and transfected with 100 ng indicated reporter plasmid and 10 ng Renilla following the recommended protocol for the Lipofectamine 2000 transfection system. After 48 h of incubation, firefly and Renilla luciferase activities in the cell lysates were measured using a dual-luciferase reporter assay system (Promega).

### Co-immunoprecipitation

Co-immunoprecipitation from the concentrated culture medium was carried out with β-catenin Dynabeads (Invitrogen) as described by the manufacturer with slight modifications. To prepare the beads for immunoprecipitation, a 40-μL bead slurry was washed twice with 200 μL PBS containing 0.005% P20 (PBSP). The anti-TCF4 antibody (Sigma) was captured on the beads by resuspending the beads in 40 μL PBSP containing 6 μL anti-TCF4 antibody and washing once in 200 μL PBSP to remove unbound antibody. Subsequently, the beads were washed twice in 200 μL PBS without P20 to wash out the P20 detergent, which may interfere with the nano-disc structure.

### RNA pulldown

RNA pulldown was performed using the RNA-Protein Pull-Down Kit (Thermo Fisher Scientific) according to the manufacturer’s instructions. Briefly, T4 RNA polymerase (Roche, Basel, Switzerland) was used to label LINC01197. Biotinylated RNAs were then mixed with streptavidin magnetic beads (Invitrogen) at 4 °C overnight. The cell lysates were reacted with biotinylated RNA on ice for 1 h. Finally, the RNA-protein binding mixtures were identified by western blot analysis.

### RNA immunoprecipitation (RIP) assay

According to the manufacturer’s instructions, an RIP assay kit (Millipore, Billerica, MA, USA) was used. Briefly, cell suspensions were prepared in RIP buffer. An anti-β-catenin antibody (Cell Signaling Technology, 5 μg) was incubated with the cell suspension at 4 °C overnight. Next, the precipitated RNA was purified and analyzed by qRT-PCR. Isotype-matched IgG (5 μg) was used as a negative control.

### Statistics analysis

Data were expressed as the means ± SEM. The unpaired, 2-tailed *t* test was used to compare 2 groups. For multiple comparisons, analysis of variance or repeated analysis of variance followed by the least significant difference post hoc test was conducted with GraphPad Prism v6.0 software (GraphPad, Inc., La Jolla, CA, USA). A *P* value < 0.05 was considered statistically significant.

## Results

### LINC01197 expression is associated with low FOXO1 expression and poor prognosis for PDAC

**Fig. 1:**
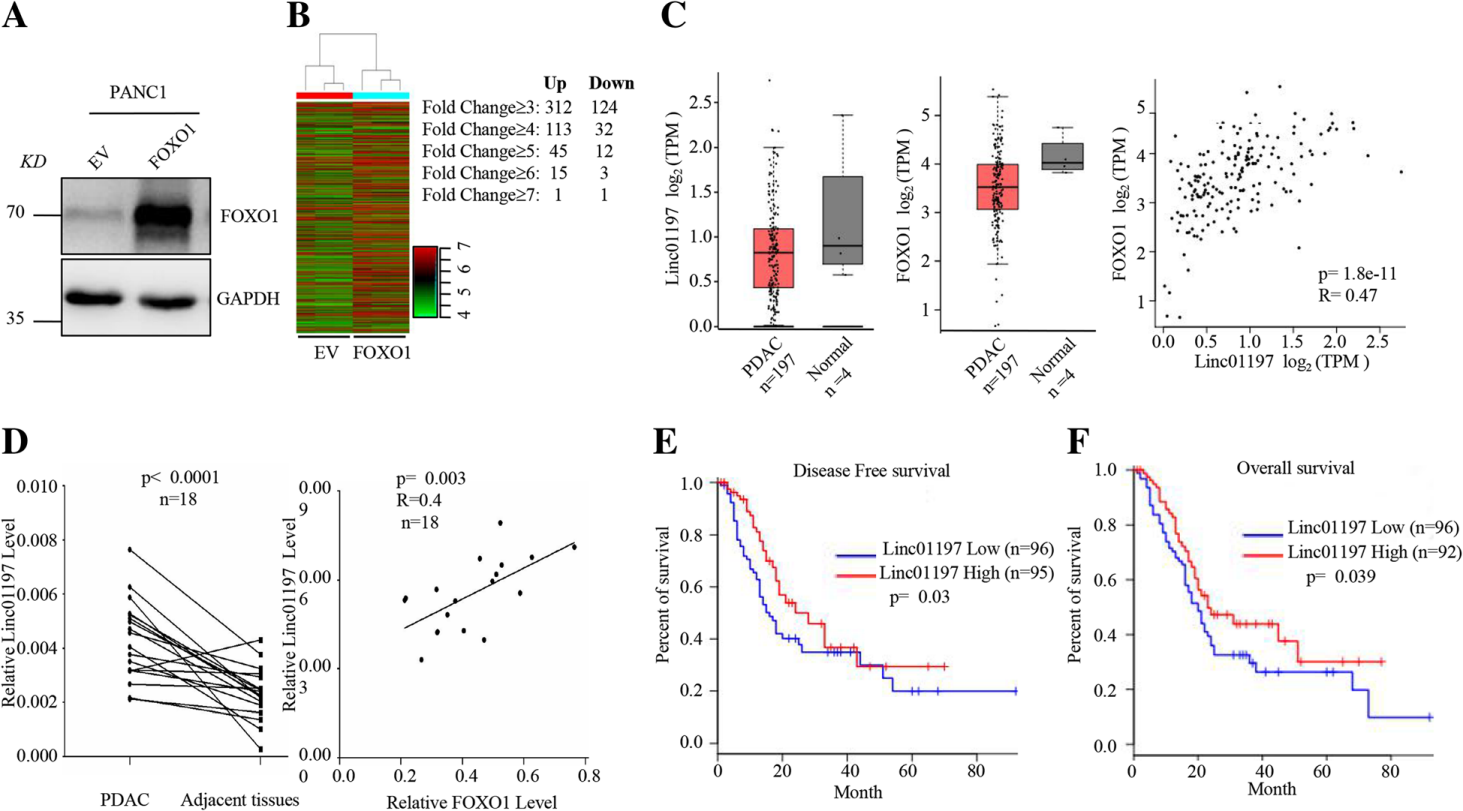
LINC01197 is positively correlated with FOXO1 and low expression predicts poor patient prognosis in PDAC. **a** FOXO1 protein level was detected by western blotting when FOXO1 overexpressed in PANC1 cells. **b** Mean centered, hierarchical clustering of genes altered in FOXO1-overexpressing PANC1 cells. **c** Data from TCGA showed that LINC01197 and FOXO1 is down-regulated in PDAC compared to in normal tissues. **d** qRT-PCR showed that expression of LINC01197 in 18 paired fresh PDAC was lowe*r* than that in adjacent tissues and positively correlated with FOXO1. **e** and **f** Data from TCGA showed that low expression of LINC01197 predicts poor disease-free survival and overall survival

Our previous study showed that FOXO1-negative cells carry cancer stem-like characteristics in PDAC [10] and affect tumor progression, suggesting that FOXO1 functions as a tumor suppressor in PDAC; however, the underlying mechanism remains unknown. We overexpressed FOXO1 in PANC1 cells (Fig. 1a) and then performed lncRNA microarray screening (Fig. 1b). FOXO1 overexpression increased the levels of 312 lncRNAs; only one lncRNA, LINC01197, was elevated by over 7-fold, suggesting its relationship with FOXO1 in PDAC. We next analyzed the expression of LINC01197 and FOXO1 in PDAC from The Cancer Genome Atlas (TCGA) and found that LINC01197 is down-regulated in PDAC tissues, as observed for FOXO1. Furthermore, the expression of LINC01197 was positively correlated with FOXO1 in the same patient cohort **(**Fig. 1c**)**. We also validated the expression of LINC01197 in 18 fresh PDAC tissues and adjacent normal tissues and found that LINC01197 was significantly down-regulated in PDAC tissues and positively correlated with FOXO1 **(**Fig. 1d**)**. These results supported that LINC01197 is regulated by FOXO1. We next analyzed the prognosis of LINC01197 in TCGA PDAC patient cohort. We found that low expression of LINC01197 predicts poor disease-free prognosis (Fig. 1e) and overall survival prognosis (Fig. 1f), demonstrating the clinical significance of LNC01197. These results suggest that LINC01197 is down-regulated in PDAC and associated with low FOXO1 expression and poor prognosis for PDAC, indicating its potential as a tumor suppressor in PDAC.

### LINC01197 is mainly localized in cell nucleus and is regulated by FOXO1

**Fig. 2:**
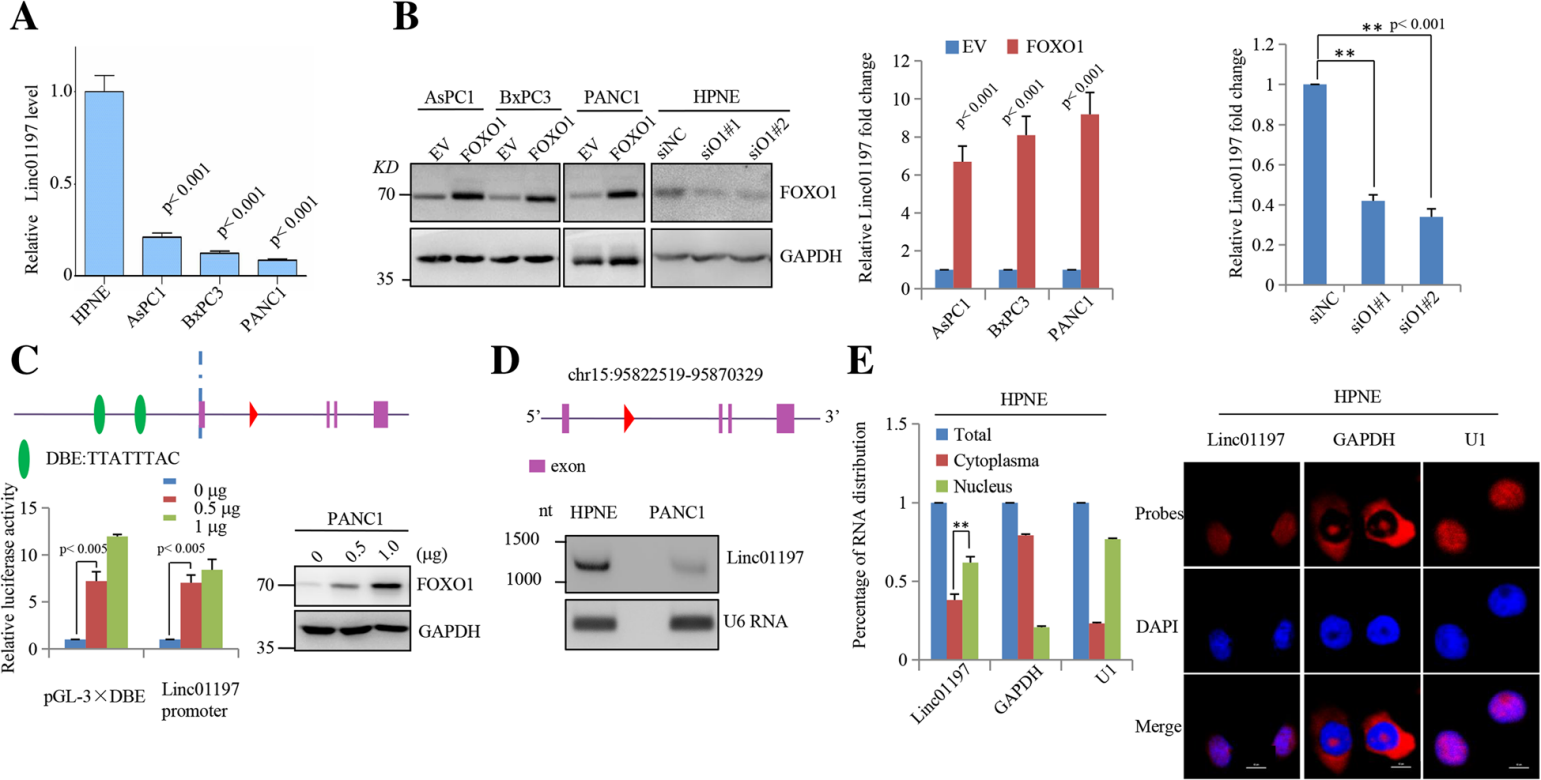
LINC01197 is a direct target of FOXO1 and is mainly localized in the cell nucleus. **a** LINC01197 expression is significantly decreased in PDAC cells compared to that in HPNE cells. **b** Altering the FOXO1 expression level similarly changed LINC01197 expression in AsPC1, BxPC3, PANC1, and HPNE cell lines. **c** Schematic representation of binding site for FOXO1 transcription factor in promoter region of the Linc01197 gene. Luciferase assay was carried out in PANC1 cells co-transfected with PGL3-3DBE or Linc01197 promoter and FOXO1 at different concentration for 48 h. **d** Northern blot assay showing LINC01197 expression level in HPNE and PANC1 cells. **e** Nucleocytoplasmic separation and FISH assays showed that LINC01197 is mainly localized in HPNE cells. *P* < 0.05 indicates a significant difference and all assays were performed in triplicate

To confirm that the expression of LINC01197 is regulated by FOXO1, we measured LINC01197 expression in the normal pancreatic ductal cell line HPNE and three PDAC cell lines and observed significant downregulation of LINC01197 in PDAC cell lines (Fig. 2a). We then overexpressed FOXO1 in AsPC1, BxPC3, and PANC1 cells and knocked down FOXO1 in HPNE cells. Overexpression of FOXO1 remarkably elevated the expression of LINC01197 in these cells. Silencing of FOXO1 in HPNE cells significantly inhibited the expression of LINC01197 **(**Fig. 2b**)**. These results support that LINC01197 is a direct target of FOXO1 in PDAC cells. We then analyzed the promoter sequence of LINC01197 and identified two FOXO1 binding sites for Daf-16 binding element(DBE) in the LINC01197 promoter region. A dual-luciferase reporter showed that FOXO1 increases the luciferase activity of the LINC01197 promoter **(**Fig. 2c**)**, demonstrating that LINC01197 is a direct target of FOXO1. LINC01197 is at chr15:95822519–95,870,329 and has a full-length of 1445 base pairs. Northern blot assays validated the presence and expression of LINC01197 in HPNE and PANC1 cells (Fig. 2d), confirming that LINC01197 expression is down-regulated in PANC1 cells. The subcellular distribution of lncRNA suggests its biological functions [28]. We found that in HPNE cells, LINC01197 was mainly localized in the nucleus (Fig. 2e). Taken together, these results demonstrate that LINC01197 is a direct target of FOXO1 and is mainly localized in the nucleus.

### LINC01197 functions as a tumor suppressor in PDAC

**Fig. 3:**
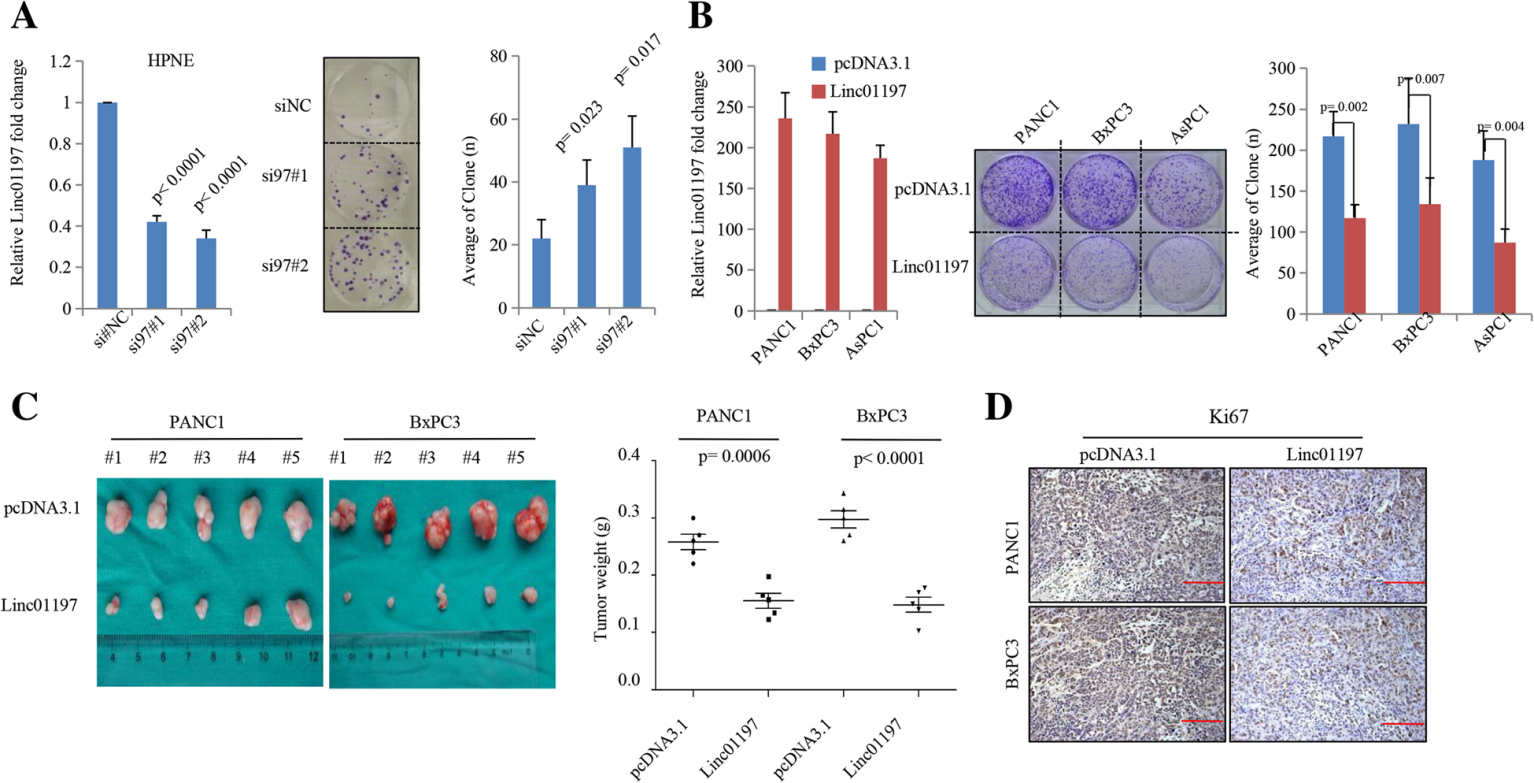
LINC01197 significantly inhibits PDAC cell proliferation and growth both in vitro and in vivo*.* **a** Clone formation assay showed that knockdown of LINC01197 dramatically increased HPNE clone numbers. **b** Clone formation ability of PDAC cell lines, AsPC1, BxPC3, and PANC1, was significantly decreased when LINC01197 was overexpressed. **c** Xenograft tumor formation assays showed that increased expression of LINC01197 inhibits subcutaneous tumor formation in PANC1 and BxPC3 cell lines. **d** IHC assays show that expression of Ki67 in PANC1 and BxPC3 stable cells induced tumor formation. *P* < 0.05 indicates a significant difference and all assays were performed in triplicate

The expression of LINC01197 is down-regulated in PDAC tissues and predictive of poor prognosis, suggesting that it plays a suppressive role in PDAC progression. We further investigated the biological functions of LINC01197. We knocked down LINC01197 in HPNE cells (Fig. 3a) and found that silencing of LINC01197 significantly elevated cell colony formation in HPNE cells (Fig. 3a). Furthermore, we overexpressed LINC01197 in PANC1, BxPC3, and AsPC1 cells and found that ectopic overexpression of LINC01197 remarkably inhibited cell colony formation in all three cell lines (Fig. 3b). To verify these effects in vivo, we performed tumor formation assays in mice. Overexpression of LINC01197 significantly inhibited the growth of PANC1- and BxPC3-derived tumors (Fig. 3c) and immunohistochemical assays showed the expression of the cell proliferation marker Ki67 [29] was remarkably down-regulated in PANC1- and BxPC3-derived tumors (Fig. 3d). Collectively, these results suggest that LINC01197 has tumor-suppressive activity in PDAC progression.

### LINC01197 binds to β-catenin and inhibits Wnt/β-catenin signaling pathway

**Fig. 4:**
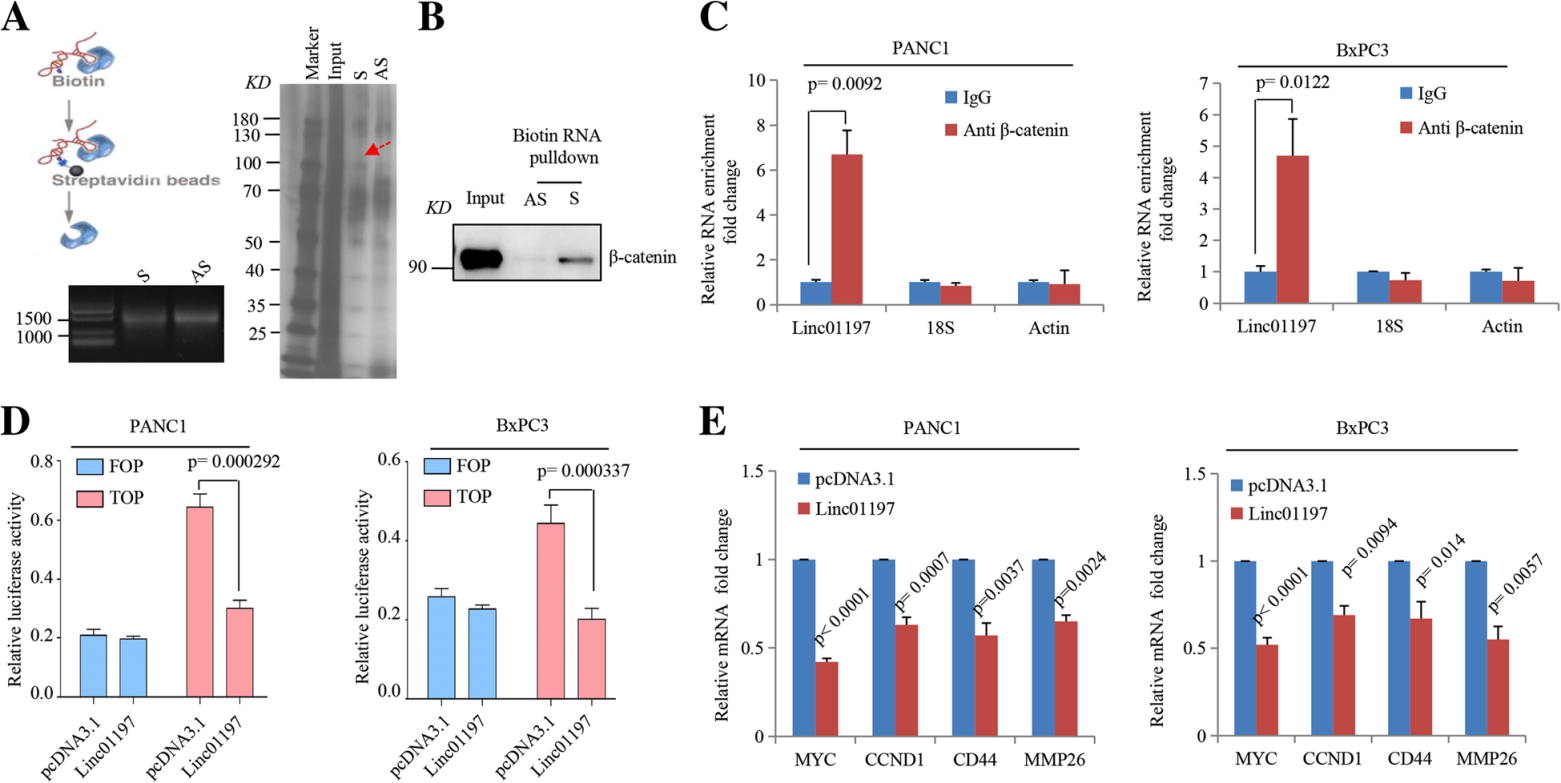
LINC01197 directly binds to β-catenin and remarkably inhibits the Wnt/β-catenin signaling pathway in PDAC cell lines. **a** and **b** Western blot of input and β-catenin complexes pulled down by LINC01197, antisense control from nuclear extracts of PANC1. **c** RIP assays showed that LINC01197 was enriched by β-catenin in indicated cell lines. **d** Dual luciferase reporter assays revealed that overexpression of LINC01197 inhibited Wnt/β-catenin signaling activity (TCF/β-catenin reporter) in PANC1 and BxPC3. **e** qRT-PCR showed MYC, CCND1, CD44, and MMP26 expression levels were significantly decreased when PANC1 and BxPC3 cells overexpressed LINC01197. *P* < 0.05 indicates a significant difference and all assays were performed in triplicate

Because LINC01197 was found to function as a tumor suppressor in PDAC, we examined its mechanism by performing biotin-labeled RNA in the PANC1 cell lysate (Fig. 4a). A potential band around 100KD was specifically enriched in the LINC01197 pull down proteins. β-catenin was identified as the most abundant LINC01197-interacting proteins via mass spectrometry, we then performed biotin-labeled RNA pulldown assays to validate the interaction between LINC01197 and β-catenin. As shown in Fig. 4b, β-catenin was significantly pulled down by LINC01197, but not by the anti-sense LINC01197-AS (Fig. 4b). β-Catenin RIP assays in PANC1 and BxPC3 cells showed that LINC01197 was significantly enriched when β-catenin was present (Fig. 4c). These results validated that LINC01197 binds to β-catenin. Considering that β-catenin plays an important role in Wnt/β-catenin signaling as a PDAC carcinogenic factor, we evaluated how LINC01197 affects Wnt/β-catenin signaling in PDAC cells. Dual-luciferase reporter assays showed that overexpression of LINC01197 robustly inhibited the luciferase activity of TOP flash in both PANC1 and BxPC3 cells (Fig. 4d). These results suggest that LINC01197 inhibits Wnt/β-catenin signaling. Evaluation of the expression of canonical Wnt target genes in PANC1 and BxPC3 cells showed that MYC, CCND1, CD44, and MMP26 expression was significantly inhibited in LINC01197-overexpressing cell lines (Fig. 4d). Taken together, these results demonstrate that LINC01197 binds to β-catenin and inhibits Wnt/β-catenin signaling.

### LINC01197 segregates β-catenin and TCF4 in PDAC cells

**Fig. 5:**
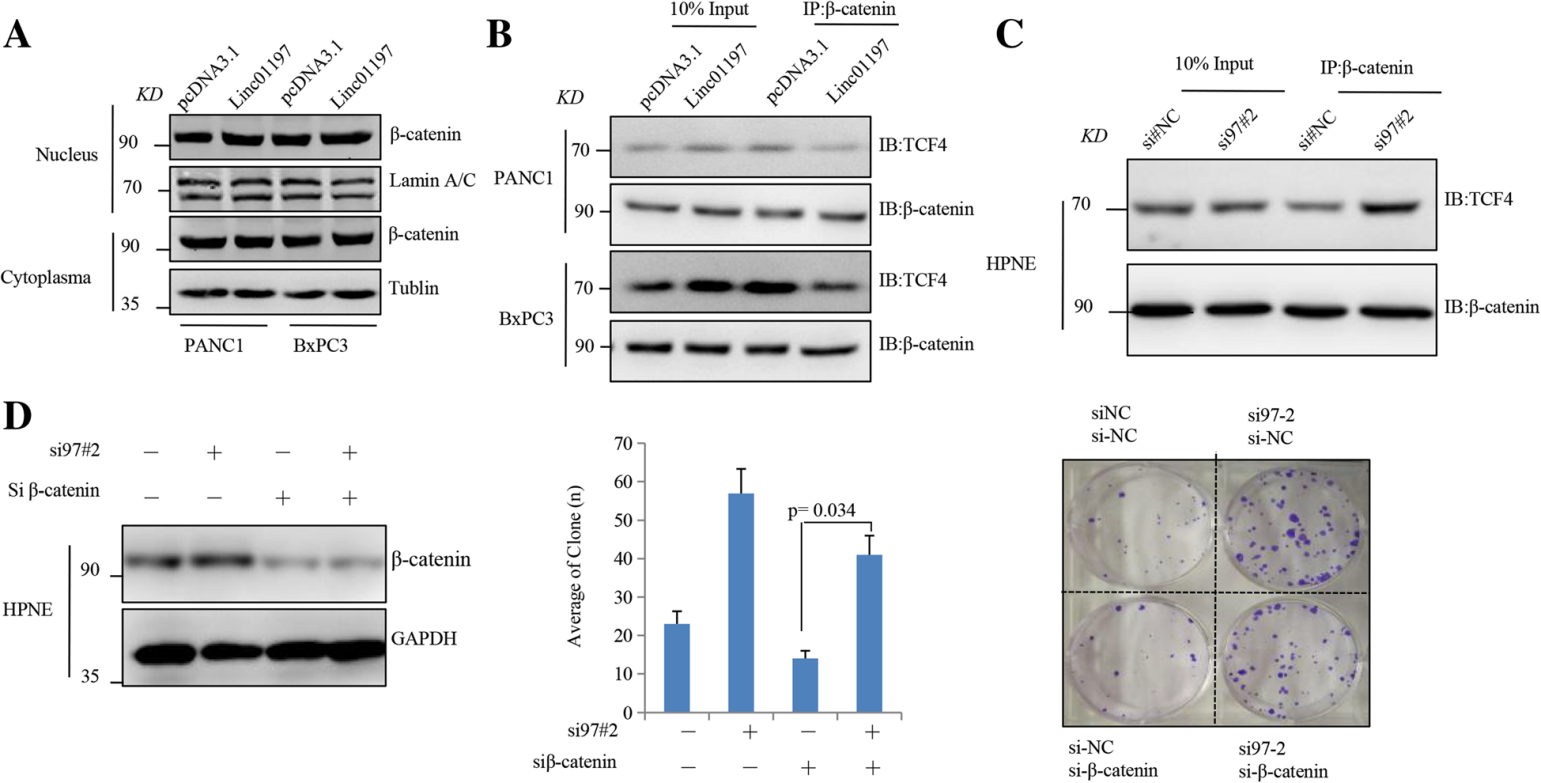
LINC01197 disrupts the interaction between β-catenin and TCF4. **a** Nuclear and cytoplasm β-catenin level as detected by western blotting in PANC1 and BxPC3 cells. **b–c** Co-IP assays showed that TCF4 binding to β-catenin is decreased in LINC01197-overexpressing PDAC cells (**B**) and significantly elevated in LINC01197-silenced HPNE cells (**c**, **d**) β-catenin absence attenuates HPNE colony formation caused by LINC01197 knockdown. *P* < 0.05 indicates a significant difference and all assays were performed in triplicate

**Fig. 6:**
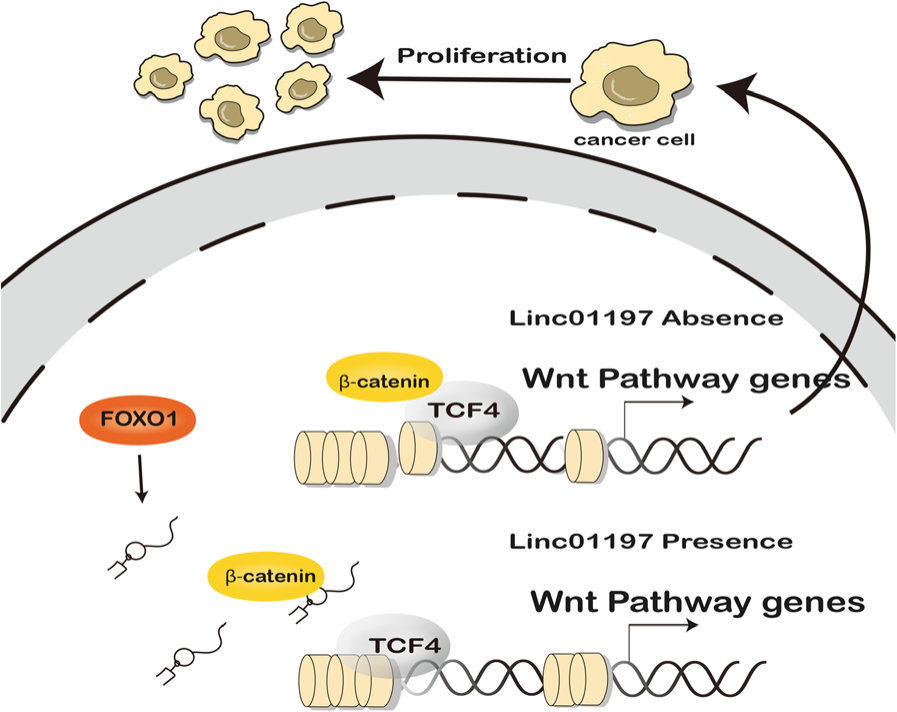
Schematic diagram for interaction between LINC01197 and β-catenin/TCF4

Because LINC01197 binds to β-catenin and inhibits Wnt/β-catenin signaling in PDAC cells, we examined whether LINC01197 inhibits the expression and nucleus translocation of β-catenin in PDAC cells. We first detected the level of β-catenin in the nucleus and cytoplasm in PANC1 and BxPC3 cells, as shown in Fig. 5a; however, ectopic expression of LINC01197 did not affect the expression and nucleus translocation of β-catenin in these two cell lines. Given that β-catenin should first bind to TCFs including TCF1, TCF4 and TCF7 [30–32] and then trigger Wnt/β-catenin signaling, we predicted that LINC01197 affects the binding of these TCFs with β-catenin in PDAC cells. Interestingly, as illustrated in Fig. 5b, we verified that overexpression of LINC01197 inhibits the binding of β-catenin and TCF4 both in PANC1 and BxPC3 cells. Additionally, silencing of LINC01197 in HPNE cells increased the binding of TCF4 to β-catenin (Fig. 5c). These results suggest that LINC01197 segregates β-catenin and TCF4 in both PDAC and normal pancreatic ductal cells. Silencing of β-catenin rescued the increased cell colony formation ability in LINC01197 knockdown HPNE cells (Fig. 5d). These results indicate that LINC01197 disrupts the interaction between β-catenin and TCF4 binding and then inhibits Wnt/β-catenin signaling in PDAC cells (Fig. 6).

## Discussion

An increasing number of studies has demonstrated that lncRNAs play oncogenic roles in tumor initiation and progression [33–35], while few tumor-suppressive lncRNAs have been identified. A previous study reported that FOXO1-related lncRNA MALAT1 can inhibit osteosarcoma cell proliferation [36], indicating the presence of a class of lncRNAs regulated by FOXO1, which play important roles in PDAC progression.

In the current study, we identified the lncRNA LINC01197 as a tumor suppressor and found that the FOXO1/LINC01197/β-catenin axis has tumor suppressor functions in PDAC. FOXO1 is a transcription factor that mainly regulates metabolic homeostasis in response to oxidative stress. In tumor biology, FOXO1 is typically down-regulated or depleted in malignant tissues in cervical cancer, ovarian cancer, renal clear cell carcinoma, and gastric cancer. Our previous study showed that FOXO1-negative cells carry cancer stem-like characteristics in PDAC [10]. In this study, we screened for FOXO1-regulated lncRNAs in PANC1 cells and found that LINC01197 was significantly down-regulated in PDAC tissues. The levels of this lncRNA were positively correlated with FOXO1, and low LINC01197 expression predicted poor prognosis for patients with PDAC. We also demonstrated that LINC01197 is directly targeted by FOXO1 in luciferase reporter assays. Dysregulation of FOXO1 disrupts metabolic homeostasis and ultimately alters cell proliferation and growth; LINC01197 shows the same effects in PDAC cells. We then characterized the biological functions of LINC01197 in PDAC cells by knocking down LINC01197 in normal pancreatic ductal HPNE cells, which increased proliferation and growth. In contrast, overexpression of LINC01197 robustly inhibited PDAC cell proliferation both in vitro and in vivo. These function demonstrate that LINC01197 is a tumor suppressor in PDAC. Localization of lncRNAs may suggest how lncRNAs exert their functions [28]. LncRNAs localized in the cytoplasm mainly function as a sponge to sequester miRNAs and their target genes [37], while lncRNAs localized in the nucleus may be involved in epigenetic regulation or guide RNA of transcription factors [38]. In this study, we found that LINC01197 is mainly localized in the nucleus but not in the cytoplasm. RNA-pulldown assays showed that β-catenin binds to LINC01197, which was confirmed by RIP assays in both PANC1 and BxPC3 cells. β-Catenin plays a pivotal role in the transduction of Wnt signaling, and dysregulation of Wnt/β-catenin signaling occurs in many types of malignant cancers, including PDAC. We further found that LINC01197 binds to β-catenin and inhibits Wnt/β-catenin signaling in PDAC cells without affecting the expression and translocation of β-catenin. Upon receiving the Wnt canonical ligand, β-catenin accumulates and is trans-localized to the nucleus where it binds to TCFs and triggers the expression of downstream target genes [20]. Numerous lncRNAs such as lncTCF7 and SNHG1 have been reported to regulate Wnt/β-catenin signaling [39, 40], while none of these lncRNAs bind to β-catenin. Our data showed that LINC01197 sequesters β-catenin binding to TCF4 in PDAC cells and then inhibits Wnt/β-catenin signaling. LINC01197 is the first lncRNA found to be involved in the β-catenin/TCF4 complex and negatively regulate the Wnt canonical signaling pathway. How LINC01197 binds to β-catenin and the clinical importance of these results should be further analyzed.

## Conclusion

In conclusion, we found that FOXO1 directly regulates an lncRNA, LINC01197, which is down-regulated in PDAC and restrains Wnt/β-catenin signaling by disrupting the interaction between β-catenin and TCF4 in PDAC. Characterization of the FOXO1/LINC01197/β-catenin axis provides important insight into PDAC prevention and treatment.

## Abbreviations

DBE: Daf-16 binding element.
FISH: Fluorescence in situ hybridization
lncRNAs: Long non-coding RNAs
PDAC: Pancreatic ductal adenocarcinoma
TCGA: The Cancer Genome Atlas
